# How Are ‘Barack Obama’ and ‘President Elect’ Differentially Stored in the Brain? An ERP Investigation on the Processing of Proper and Common Noun Pairs

**DOI:** 10.1371/journal.pone.0007126

**Published:** 2009-09-23

**Authors:** Alice Mado Proverbio, Serena Mariani, Alberto Zani, Roberta Adorni

**Affiliations:** 1 Department of Psychology, University of Milano-Bicocca, Milan, Italy; 2 Institute of Bioimaging and Molecular Physiology, National Research Council (CNR), Milano-Segrate, Italy; Victoria University of Wellington, New Zealand

## Abstract

**Background:**

One of the most debated issues in the cognitive neuroscience of language is whether distinct semantic domains are differentially represented in the brain. Clinical studies described several anomic dissociations with no clear neuroanatomical correlate. Neuroimaging studies have shown that memory retrieval is more demanding for proper than common nouns in that the former are purely arbitrary referential expressions. In this study a semantic relatedness paradigm was devised to investigate neural processing of proper and common nouns.

**Methodology/Principal Findings:**

780 words (arranged in pairs of Italian nouns/adjectives and the first/last names of well known persons) were presented. Half pairs were semantically related (“Woody Allen” or “social security”), while the others were not (“Sigmund Parodi” or “judicial cream”). All items were balanced for length, frequency, familiarity and semantic relatedness. Participants were to decide about the semantic relatedness of the two items in a pair. RTs and N400 data suggest that the task was more demanding for common nouns. The LORETA neural generators for the related-unrelated contrast (for proper names) included the left fusiform gyrus, right medial temporal gyrus, limbic and parahippocampal regions, inferior parietal and inferior frontal areas, which are thought to be involved in the conjoined processing a familiar face with the relevant episodic information. Person name was more emotional and sensory vivid than common noun semantic access.

**Conclusions/Significance:**

When memory retrieval is not required, proper name access (*conspecifics knowledge*) is not more demanding. The neural generators of N400 to unrelated items (unknown persons and things) did not differ as a function of lexical class, thus suggesting that proper and common nouns are not treated differently as belonging to different grammatical classes.

## Introduction

It has been claimed that proper and common nouns are differentially implemented in the brain. It is not completely understood, however, if common/proper differences are due to differences in storage or representation of lexical knowledge [1]–[2] or differences in processes such as memory retrieval [3]–[4].

The first evidence of dissociation in the processing of proper vs. common nouns came from neurological observations of a specific impairment in the retrieval or processing of one or the other category (see table 1 for some of the more relevant studies reporting category-specific anomia cases). However, the relative heterogeneity of the lesioned area involved in these clinical cases does not allow a definitive conclusion to be reached about the existence of a distinct neural representation of a person's conceptual knowledge, in the form of independent lexical storage.

**Table 1:** Clinical studies reporting a specific dissociation between category-specific anomia cases.

Authors	Impaired name category (ANOMIA)	Lesioned area
Carney & Temple, 1993 [46]	Person names	Multiple
Cipolotti et al., 1993 [47]	Objects. (Preserved: Person and countries names)	Left fronto-parietal and thalamus
Cohen et al.,1994 [48]	Person names	Left thalamus
Fery et al., 1995 [49]	Person names	Slight left cerebral atrophy and internal capsule
Harris & Kay, 1995 [50]	Person names	Left temporal
Hittmair-Delazer et al.,1994 [30]	Person names	Left fronto-temporal including basal ganglia
Kay and Hanley, 2002 [51]	Objects, animals (Preserved: person names)	Left hemispheric infarct
Lucchelli & De Renzi, 1992 [52]	Person names	Left thalamus
Lyions et al., 2002 [53]	Objects, geographical names (Preserved: person names)	Left frontal lobe
Martins & Farrajota, 2007 [31]	Things (Preserved proper names)	Insula, parietal lobe, the temporal neocortex, including the temporal pole
Martins & Farrajota, 2007 [31]	Person names	Left infero-medial temporo-occipital cortex sparing the temporal pole
McKenna e Warrington, 1980 [32]	Person names	Left Temporal
McKenna & Warrington, 1978 [54]	Body parts, animals, objects (Preserved: places names)	Left temporal and parietal lobes
Miceli et al., 2000 [9]	Person names	Left temporal
Otsuka et al., 2005 [55]	Person names	Left Superior temporal gyrus
Semenza & Sgaramella, 1993 [56]	Preserved: Person names	Left occipito/temporal
Semenza & Zettin, 1988 [36]	Persons and geographical names (Preserved: body parts, fruits, vegetables, vehicles, pasta, furniture, colours).	Left occipito-parietal
Semenza & Zettin, 1989 [37]	Person and geographical names	Left fronto-temporal
Thompson et al., 2003 [57]	Animals, foreign animals, birds and fruit; artefact categories (preserved person names)	Right temporal lobe atrophy
Warrington & Clegg, 1993 [58]	Preserved: Place names	Left temporal cortical atrophy

To make things worse, neuroimaging studies have provided somewhat conflicting evidence, for example, the PET study by Gorno Tempini and coworkers [5] showed that the left superior temporal gyrus, the left angular and sovramarginal gyrus, and the posterior medial temporal lobe were involved in processing proper names, whereas the PET study by Campanella et al. [6] showed that the inferior frontal and inferior parietal cortex have a role in providing an association between familiar faces and names. Douville and coworkers [7] demonstrated the crucial involvement of the hippocampal and medial temporal cortex in the processing of famous names, whereas the classical paper by Damasio and coworkers [8] showed with both neurometabolic and clinical evidence that the temporal pole has a prominent role in retrieving proper names.

Overall, and notwithstanding the abundance of clinical cases of proper/common name dissociations in anomia, not many neuroimaging (except for ERP) studies have directly compared processing of proper/common nouns. The electrophysiological studies by Dehaene and coworkers [2] and Proverbio and coworkers [4] found a larger left temporal (250 ms) negativity to proper than common nouns, in a word classification and a memory retrieval task, respectively. On the basis of such a scarce evidence it is difficult to establish if proper/common names differences are based on their belonging to different semantic categories (for example, *person knowledge* [1], [9] vs. object knowledge) or on a difference in memory retrieval processes.

While, for example Douville et al. [7] did compare processing of familiar vs. unfamiliar persons, whose names were provided to participants and no memory retrieval was required, however, no comparison was made with common name processing in this fMRI study. On the other hand, proper name anomia cases rely on the evidence of patients unable to recalling the names of familiar people on the basis of their picture, or to recall biographical information about people on the basis of their name, thus, in both cases, on memory retrieval impairment.

Overall, it seems that the memory retrieval of proper names is more demanding than that of common nouns. One example of this observable fact is given by the so-called *tip-of-the-tongue* phenomenon, when it is more difficult to access the phonological forms of people's names than common nouns [10], [11], [12]. The reason relies on the fact that proper names are purely referential expressions with totally arbitrary phonological forms, whereas common nouns are often organized semantically in terms of common roots (e.g., polarity, polarization, Polaroid, pole, polestar, pole vault). Several models have been proposed to explain this category-related difference across name classes. For examples, the representational model by Cohen [13] postulates that the difficulty in retrieving proper names lies at the level of processing rather than storage. Indeed, person names are usually semantically neutral and offer little semantic depth to aid retrieval.

An electrophysiological study has specifically questioned whether the difference between proper and common noun classes consists in their belonging to two different semantic domains (or grammatical classes) or to a difference in the memory retrieval processes [4]. In that study, participants were presented with short, written, unequivocal definitions of common and proper nouns (i.e., eatables, animals, vegetables and fruits, natural events, professions, places, medical concepts, things, vs. last names of politicians, artists, historical personages, geographical names), while the task consisted in silently retrieving the defined names in order to perform a phonological decision task Overall, the retrieval of proper names was more demanding, accompanied by slower RTs and a stronger fronto-central activity than the retrieval of common nouns. Furthermore, proper name retrieval was linked to an anterior temporal activity (neatly consistent with the findings of Damasio et al. [8]), while common noun retrieval engaged the occipital areas to a greater extent, probably because more visual–sensory associations are linked to the names of highly imaginable objects (as opposed to the numerous abstract geographical names). The data were interpreted as indicating the activation of partially overlapping cortical regions differentially involved in memory retrieval because of the specific properties of the two lexical categories. Person name retrieval was compared to recall of episodic information, characterized by complex contextual properties and very precise spatio-temporal coordinates, whereas common nouns were compared to the retrieval of more redundant and distributed information. The aim of the present study was to investigate the way in which different semantic domains are represented in the brain without the involvement of memory processes.

In order to investigate the neural processing of proper vs. common nouns, thus gaining information about how conceptual and episodic knowledge is stored in the brain, 380 names in the two categories arranged in well- or ill-assorted noun/adjective pairs (in Italian this is the correct order) and first/last names (for a total number of 780 stimuli) were visually presented to the participants; therefore memory retrieval of names (which is thought to be more effortful for purely referential expressions [11], [12], [13]) was not required. The task consisted in deciding whether the second item of each pair was semantically related to the first; all items were balanced for a series of linguistic and perceptual factors within and across classes.

The uniqueness of the present study, in our view, is that we included comparisons of both familiar and unfamiliar common/proper nouns in the same design, and that both proper and common unfamiliar nouns were created by randomly mixing sets of real first/last names and adjective/noun word pairs, to balance for semantic associativeness. Furthermore, names were directly provided to the reader and did not need to be retrieved by subjects.

We assumed that unrelated word pairs (either common or proper nouns) elicited a centro-parietal negativity, know as N400 component (N400 paradigm, [14], [15]), thought to reflect the amount of effort needed to semantically integrate the incoming word into the previous semantic context (e.g., [16]) and/or the ease or difficulty of retrieving stored conceptual knowledge associated with a word [15], [17]. On the other hand, for related second words of a pair we expected the eliciting of standard P3 component (which appears each time the task requires a binary-type decision [18], and which peaks at about 400 ms at central sites (e.g., [19]). While not specifically sensitive to language, P300 will be elicited in any psycholinguistic paradigm that requires stimulus evaluation and a binary decision. The amplitude of the P300 is believed to vary with the participant's confidence in their decision, and its latency would index when the decision is made. This positive deflection may overlap in time with more specifically language-sensitive ERP component (i.e., N400, [20]), thus evidencing a P/N400 modulation.

One of the hypotheses was that if the two set of stimuli differed because they belonged to different semantic domains, this factor, in principle, would affect the processing of both related and unrelated pairs. The contrast between related and unrelated items was devised to reveal the neural structures devoted to processing linguistic contents related to persons and to common entities. More specifically, we hypothesized that any difference in N400 amplitude in response to unrelated items of the two categories, or in the underlying neural generators investigated by means of LORETA source reconstruction, would suggest a differentiated neural mechanism for representing words belonging to the two categories. On the contrary, a lack of it would support the alternative hypothesis that proper/common nouns differences might be related to memory retrieval processes [3]–[4] and perhaps their affinity with episodic vs. semantic information. Furthermore, since proper and common names were matched for many perceptual and linguistic factors (except for imagery value, which was measured a posteriori), we expected larger P400 responses and faster RTs to nouns easier to be semantically accessed and evaluated.

## Methods

### Participants

Sixteen healthy right-handed Italian University students (7 males and 9 females) were recruited for this experiment. Their ages ranged from 20 to 35 years (mean = 24.9 years; SD = 3.5). All had normal or corrected-to-normal vision and reported no history of neurological illness or drug abuse. Their handedness was assessed by the Italian version of the Edinburgh Handedness Inventory, a laterality preference questionnaire reporting strong right-handedness and right ocular dominance in all participants. Experiments were conducted with the understanding and the written consent of each participant. The experimental protocol was approved by the ethical committee of the National Research Council in Milan. Two subjects were subsequently discarded because of excessive EOG artefacts.

### Stimuli

One hundred and ninety pairs of proper names (consisting of a first name followed by a last name) of famous people, and 190 pairs of common nouns (comprising a noun with a strongly related adjective), were used as stimuli in the present experiment (see the complete list of 380 word pairs in Appendix S1). They were randomly presented at the centre of a PC screen located about 114 cm from the viewer's eyes. Stimulus duration was 150 ms for both the first and second element in each pair, which were presented in sequence with an ISI varying between 650 and 750 ms. The inter-trial interval (ITI) varied between 1500 and 1600 ms. Words were written in capital letters and Arial Narrow font. They were white on a gray background, 36′ 13″ (1.2 cm) in height and from 1° 15′ 27″ to 6° 32′ 20″ (2.5 to 13 cm) in length.

#### Selection of common nouns

Common noun pairs were selected from a wider set of 240 strongly associated nouns and adjectives that were randomly presented to a preliminary group of 18 judges (5 men and 13 women, 18–35 years of age). The judges were required to evaluate the semantic associativeness of each noun-adjective pair on a 5-point scale. The 190 word pairs were selected if they gained an average semantic associativeness of 4.45 on this procedure. They were then subdivided into two groups of 95 semantically related and 95 semantically unrelated pairs, the latter obtained by mixing adjectives randomly with unrelated nouns. Items belonging to the two groups (related and unrelated) were balanced for length and written frequency of use. Since ERPs were time-locked to the onset of the second item in each pair (the adjective), specific balancing was performed on the adjective. Their frequency of use was measured according to the COLFIS dictionary [21]. This corpus comprises 3,798,275 words from contemporary written Italian texts, and represents the Italian texts that are actually read rather than all possible written texts. It includes 1,836,119 entries taken from the most popular newspapers, 1,306,653 from periodicals and 655,503 from books.

#### Balancing of common nouns

The lengths were 7.61 letters for related and 7.59 for unrelated pairs. Frequency of use was 39.99 for semantically related and 37.51 for semantically unrelated pairs. Two one-way ANOVAs were performed on length (F(1, 188) = 0.00669, p = 0.935) and frequency values (F(1, 188) = 0.0639, p = 0.801) across the two groups of semantically related and unrelated pairs of common nouns, respectively. This demonstrated a substantial balancing of the two factors.

#### Selection of proper names

Proper name pairs were selected from a wider set of 240 first and last names of internationally famous people (mostly Italian) belonging to show business or to the political, science, arts or sport worlds, mostly contemporary. All selected personages were quite popular at the time of EEG recording (spring 2008). Only persons whose first and last names were highly familiar as semantically related names were selected. People known only by their first or last names were not included (e.g. the singer Sting). In order to select the best stimuli for our experimental purpose, the familiarity of the names and their degrees of forename-surname associativeness were measured by administering two questionnaires to a preliminary group of 18 judges (5 men and 13 women, 18–35 years of age). The order of administration was counterbalanced across individuals. To assess the familiarity of the names, the judges were required to evaluate popularity according to their subjective experience using a 5-point scale (going from highly familiar to unknown).

The 190 word pairs were selected if they gained an average semantic associativeness of 4.45 on this procedure. They were then subdivided into two groups of 95 semantically related and 95 semantically unrelated pairs, obtained by mixing adjectives randomly with unrelated nouns. Items belonging to the two groups (related and unrelated) were balanced for length and written frequency of use. For first/last name associativeness, judges were asked to evaluate the extent to which the first and last names of each personage were associated using another 5-point scale.

On the basis of this procedure, two subgroups of name pairs were formed. The first comprised 95 names characterized by a high degree of familiarity for all subjects (4.50) and a high degree of first/last name associativeness (4.62). The second comprised 95 pairs of names of equal (initial) familiarity but low associativeness, and were obtained by randomly mixing the first names of famous people with the last names of others (see some examples in Appendix S1).

#### Balancing of proper names

The resulting pairs were therefore balanced for length and frequency of use. Since the ERPs were time-locked to the onset of the second item of each pair (the surname), the balancing was performed only for last names. Their lengths were respectively 7.61 letters for related and 7.59 for unrelated pairs. Frequency of use was 31.56 for semantically related and 32.87 for semantically unrelated pairs. Two one-way ANOVAs were performed on length (F(1, 188) = 0.0081; p = 0.9285) and frequency values (F(1, 188) = 0.0217; p = 0.8831) across the two groups of semantically related and unrelated pairs of proper names, respectively, This demonstrated a substantial balancing of the two factors.

#### Balancing between common and proper noun classes

Overall, on the basis of the previous balancing procedure, 380 pairs of nouns were selected, half belonging to the proper name category and the other half to the common noun category. Half of each group formed a pair of semantically related items (either proper or common nouns) and the other half a pair of unrelated items (either proper or common nouns). The two classes were balanced for length (common nouns = 7.60; proper names = 7.34; these values were statistically identical (F(1,378) = 2.186; p = 0.1401) and written frequency of use (common nouns = 38.75; proper names = 32.22; these values were also statistically identical: F(1, 378) = 0.966; p = 0.3264). The related pairs of common and proper nouns were also comparable in degree of semantic associativeness (common nouns = 4.42; proper names = 4.59).

### Task and procedure

The participants, seated comfortably in a dimly lit, electrically and acoustically shielded room, faced a window behind which a high resolution VGA computer screen was positioned 114 cm from their eyes. A small bright dot (1 mm in size) located at the centre of the screen served as a fixation point to minimize eye movement. The subjects were instructed to fixate the centre of the screen and to avoid any eye or body movement during the recording session. The task consisted in deciding whether or not the target word was semantically associated with the previous item (practically, to determine if the word pairs defined an existing known person or a familiar existing thing), by pressing one button as accurately and rapidly as possible with the index finger or middle finger to signal a yes or no response, respectively. The two hands were used alternately during the recording session, and the hand and sequence order were counterbalanced across subjects. The experimental session was preceded by two novel sequences of training. The beginning of each trial was preceded by three visually presented warning signals (Attention! Set! Go!).

### EEG recording and analysis

The EEG was continuously recorded from 128 scalp sites at a sampling rate of 512 Hz. Horizontal and vertical eye movements were also recorded. Linked ears served as the reference lead. The EEG and electro-oculogram (EOG) were amplified with a half-amplitude band pass of 0.016–100 Hz. Electrode impedance was kept below 5 kΩ. EEG epochs were synchronized with the onset of stimulus presentation and analyzed by ANT*-EEProbe* software. Computerized artefact rejection was performed before averaging to discard epochs in which eye movements, blinks, excessive muscle potentials or amplifier blocking occurred. EEG epochs associated with an incorrect behavioural response were also excluded. The artefact rejection criterion was peak-to-peak amplitude exceeding 50 µV, and the rejection rate was ∼5%. ERPs were averaged off-line from -100 ms before to 1000 ms after stimulus onset.

The mean area amplitude of P/N400 was measured at posterior sites (occipito/temporal and temporo/parietal PPO1, PPO2, TP7, TP8) in the time windows 300–380 ms and 380–500 ms. The mean amplitude of the P/N400 component was also measured at anterior sites (CP1, CP2, FC1, FC2) between 300 and 380 ms and between 380 and 500 ms. N400 peak latency was also measured for ERP to unrelated proper and common nouns at occipito/temporal (PP01, PP02), central (C1, C2) and fronto/central (FC1, FC2) sites in the time window 250–500 ms of post-stimulus latency. The choice of the two time-windows was based on post-hoc visual inspection. The first window corresponded to the peak of the positive response (P400) to associated word pairs, whereas the second one corresponded to the peak of the negative deflection (N400) to unrelated word pairs. P/N400 deflections were symmetrically measured at posterior and anterior sites where they reached their maximum amplitude to investigate possible topographical and functional dissociations.

Response times exceeding mean ±2 standard deviations were excluded. Behavioural and ERP data were subjected to multifactorial repeated-measures ANOVAs. The factors were “lexical class” (proper, common), “response hand” (left, right) and “associativeness” (associated, not associated) for RT data, and in addition, “electrode”, (dependent on ERP component of interest) and “hemisphere” (left, right) for ERP data. Multiple comparisons of means were done by post-hoc Tukey tests.

Topographical voltage maps of ERPs were made by plotting colour-coded isopotentials obtained by interpolating voltage values between scalp electrodes at specific latencies. *Low Resolution Electromagnetic Tomography* (LORETA [22]) was performed on the ERP difference waves of interest at various time latencies using *ASA4* software. LORETA, which is a discrete linear solution to the inverse EEG problem, corresponds to the 3D distribution of neuronal electric activity that has maximum similarity (i.e. maximum synchronization), in terms of orientation and strength, between neighbouring neuronal populations (represented by adjacent voxels). Source space properties were: grid spacing = 5 mm; estimated SNR = 3. In this study an improved version of standardized sLORETA was used, which incorporates a singular value decomposition-based lead field weighting: swLORETA [23].

## Results

### Behavioural data

Response times (RTs) were faster when the right hand (728 ms) rather than the left hand (740 ms) was used, as indicated by hand significance (F(1,13) = 5.08, p<0.05). They were also shorter in response to proper (717 ms) than common (752 ms) nouns, as shown by lexical class significance (F(1,13) = 47.19, p<0.01). Semantic associativeness was also significant (F(1,13) = 93.44, p<0.01), with faster RTs in response to associated (687 ms) than non-associated (781 ms) items in a pair. Error and omission rates were very low, with an average of 0.1 omissions per subject and 1.5 errors per subject.

### Electrophysiological data

**Figure 1:**
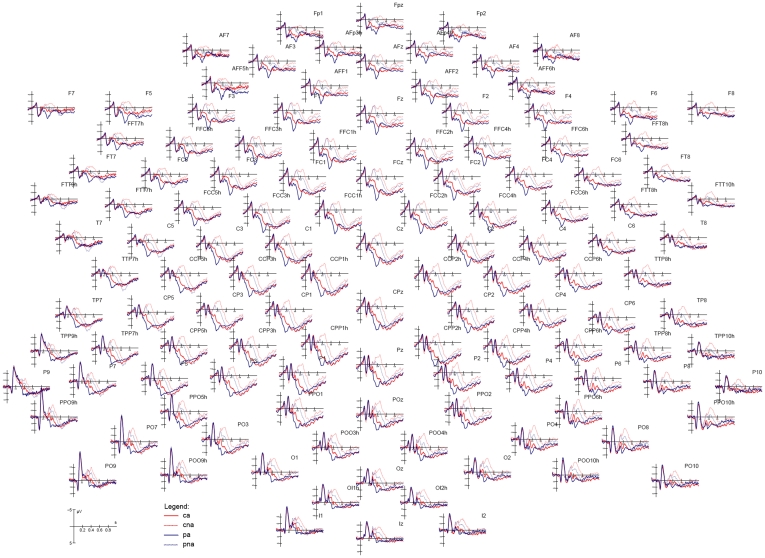
Grand-average ERP waveforms (N = 14) recorded over all scalp sites as a function of stimulus class (Key. CA = common nouns associated, CAN = common nouns unrelated, PA = proper names associated, PNA = proper names unrelated).

**Figure 2:**
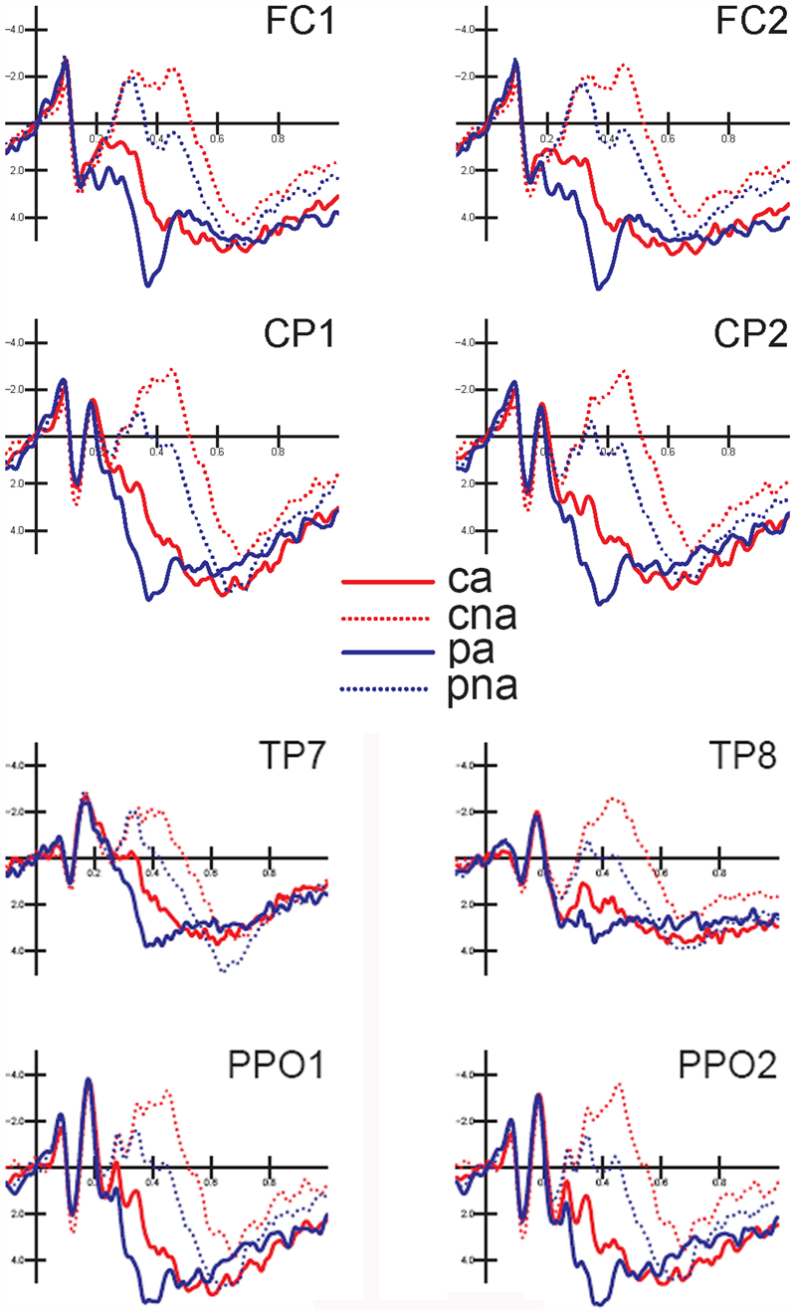
Grand-average ERP waveforms (N = 14) recorded over the occipito/temporal, temporo/parietal, centro/parietal and fronto/central sites, as a function of stimulus type.

Fig. 1 shows the grand-average waveforms (N = 14) recorded in response to the four noun categories over all scalp sites. Strong effects of both semantic associativeness and lexical class are evident after 300 ms post-stimulus latency, over several scalp sites, as illustrated in Fig. 2, showing the electrode sites selected for P/N400 measurements at posterior areas.

#### Occipito/temporal P/N400 (300–380 ms)

**Figure 3:**
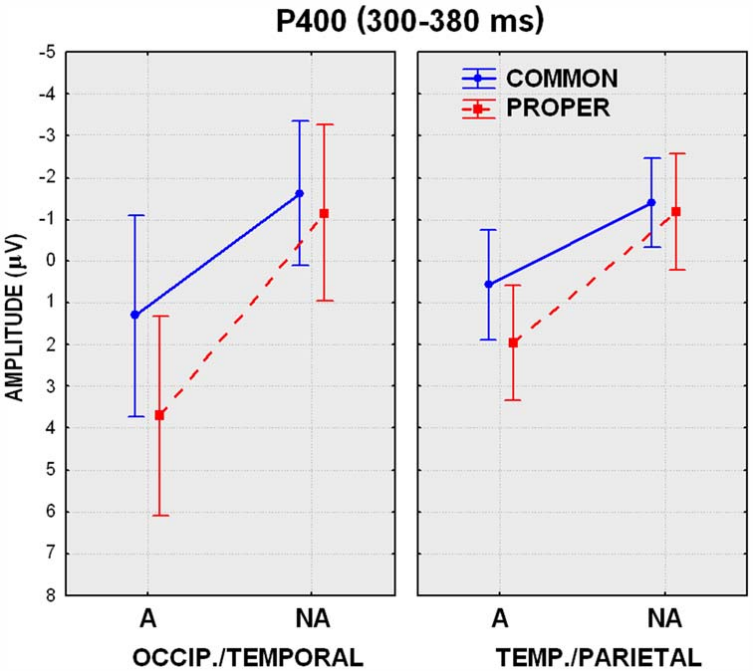
Mean amplitude values of P/N400 measured at the posterior sites in the 300–380 ms time-window.

ANOVA on the P/N400 amplitudes recorded at the PPO1-2 and TP7-8 electrode sites showed an effect of lexical class X semantic associativeness (F(1,13) = 12.00, p<0.01). Post-hoc comparisons indicated greater P400 responses (p<0.01) to related proper (2.83 µV) than common (0.95 µV) nouns, while there was no difference between unrelated nouns in the two classes (see amplitude values in Fig. 3). The further interaction of lexical class X electrode (F(1,13) = 32.01, p<0.01) indicated a greater effect of lexical class at occipito/temporal than temporo/parietal sites (with a much greater P400 to proper names at the former than the latter site), and no electrode effect for potentials elicited by common nouns. The interaction of associativeness and electrode (F(1,13) = 32.05, p<0.01) demonstrated the existence of a stronger semantic relatedness effect at the occipito/temporal site (with larger P400 to related than unrelated items) and a lack of electrode effect for unrelated items. The triple interaction of lexical class x semantic associativeness x electrode (F(1,13) = 8.47, p<0.05) confirmed a lack of electrode or lexical class effects for unrelated items and the presence of a much larger P400 to related proper (3.71 µV) than common (1.32 µV) nouns at occipito/temporal sites (see Fig. 3).

#### Occipito/temporal P/N400 (380–500 ms)

In the next temporal window, P/N400 showed that lexical class x electrode was significant (F(1,13) = 9.03, p<0.05), with larger P400 to proper than common nouns, especially at occipito/temporal sites. The further interaction semantic relatedness x electrode (F(1,13) = 32.02, p<0.01) confirmed the previous pattern of a lack of topographic effect for unrelated items (Tukey test, p = 0.43), along with larger P400 to related than unrelated items at occipito/temporal sites.

#### N400 latency

The latency of the N400 response to unrelated items was measured in the time window 250–500 ms at the FC1, FC2, C1, C2, PPO1 and PPO2 electrode sites. ANOVA showed that lexical class (F(1,13) = 6.94, p<0.05) and hemisphere (F(1,13) = 6.16, p<0.05) were significant. N400 was earlier in response to proper (340 ms) than common (380 ms) nouns and over the left (350 ms) than the right (370 ms) hemispheres.

#### Fronto-central P/N400 (300–380 ms)

**Figure 4:**
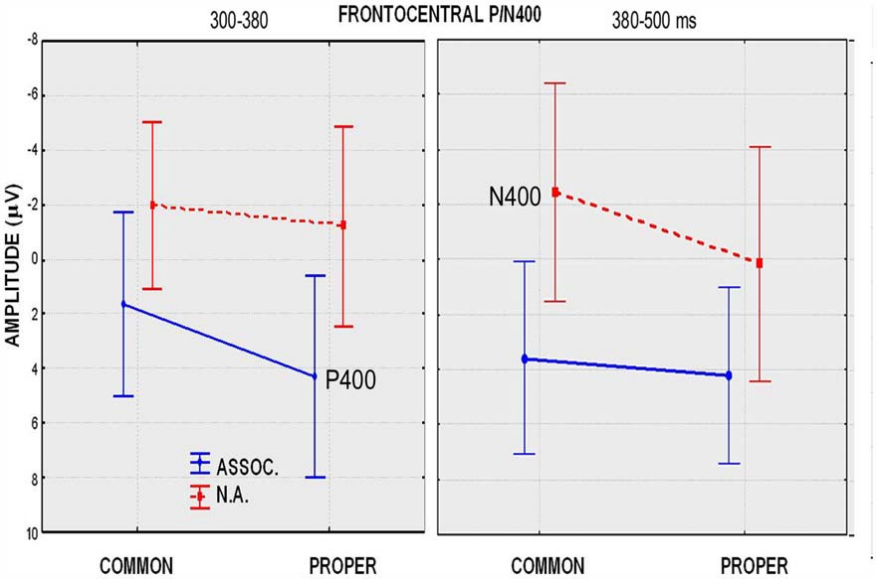
Mean amplitude values of P/N400 measured at the fronto/central site in the 300–380 and the 380–500 ms time-windows.

ANOVA performed on the P/N400 amplitude values recorded at the anterior dorsal (CP1, CP2, FC1, FC2) sites between 300 and 380 ms showed that lexical class x associativeness was significant (F(1,13) = 7.25, p<0.05). Relative post-hoc comparisons demonstrated larger P400 responses to related pairs of proper (4.27 µV) than common (1.66 µV) nouns, and no class differences in the N400 response to unrelated pairs (see left part of Fig. 4). The ANOVA also showed that semantic associativeness x electrode x hemisphere was significant (F(1,13) = 10.56, p<0.01), with a larger N400 to unrelated items over the left centroparietal area (CP1 = −1.55 µV, CP2 = −0.99 µV; Tukey test. p<0.01).

**Figure 5:**
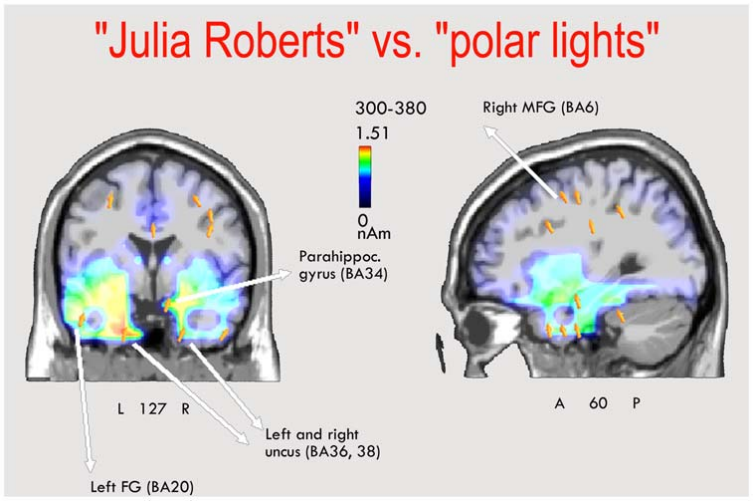
Coronal and sagittal views of active sources for the contrast-related Proper – Common nouns in the 300–380 ms time window, according to swLORETA.

In order to locate the possible neural source of the lexical effect for related words, a swLORETA source reconstruction was performed on the difference-wave obtained by subtracting the ERPs to related common nouns from those elicited by related proper names in the time window 300–380 ms. The resulting neural activity might represent a P3 response indicating the recollection of famous people as opposed to familiar things. The inverse solution showed that the processing of names of famous personages was associated with stronger activity in a series of left and right hemispheric regions, listed in Table 2, including affective and memory regions such as the parahippocampal gyrus, uncus and cingulate cortex, and face-specific regions such as the left fusiform gyrus of the temporal cortex and the right medial temporal gyrus, as also visible in Fig. 5.

**Table 2:** Recognized Persons – Things.

Magn.	T-x	T-y	T-z	Hem.	Area
1.51	−18.5	−8	−28.9	LH	Limbic lobe, Uncus, BA 36
1.39	11.3	−9.4	−14	RH	Limbic lobe, Parahippocampal gyrus, BA34
1.36	21.2	9.1	−27.5	RH	Limbic lobe, uncus, BA 38
1.18	−48.5	−33.7	−23.6	LH	Temporal lobe, Fusiform gyrus, BA20
1.12	50.8	−0.6	−28.2	RH	Temporal lobe, Medial temporal gyrus, BA21
0.94	1.5	−20.3	26.8	RH	Limbic lobe, cingulate gyrus, BA 23
0.56	40.9	2.4	29.4	RH	Frontal lobe, Inferior frontal gyrus, BA6
0.49	40.9	−40.6	34	RH	Parietal lobe, supramarginal gyrus, BA40
0.48	31	−7	46.3	RH	Frontal lobe, medial frontal gyrus, BA6
0.39	−28.5	−14.4	45.5	LH	Frontal lobe, precentral gyrus, BA4

Tailarach coordinates (mm) corresponding to the intracranial generators explaining the difference voltages related to Proper–Common nouns in the 300–380 ms time window, according to swLORETA (ASA) [23], grid spacing = 5 mm, estimated SNR = 3.

**Figure 6:**
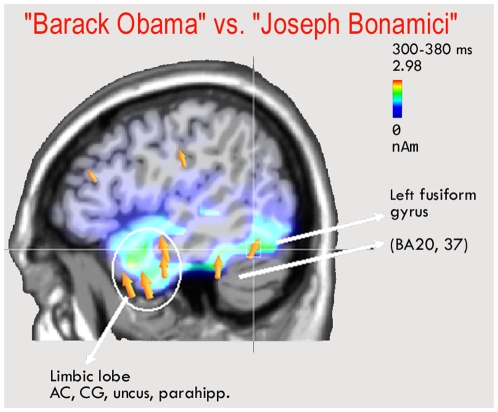
Sagittal view of active sources for the contrast-related minus unrelated proper names, according to swLORETA, in the time window 380–380 ms.

A further LORETA was performed in the same temporal window (300–380 ms) but considering the difference-waves obtained by subtracting ERPs to unrelated proper names from those to related proper names. The resulting neural activity, visible in Fig. 6, might represent a P300 response indicating the recollection of famous people as opposed to unknown people. Table 3 lists the significant sources of bioelectrical activity, which included extensive limbic and hippocampal areas (BA23,24,28,34,38), the left fusiform gyrus of the temporal cortex (BA20 and 37), the left and right medial temporal gyrus (BA20 and 21), and the inferior frontal (BA6) and inferior parietal areas (BA40).

**Table 3:** Famous persons – unknown people.

Magn.	T-x	T-y	T-z	Hem.	Area
2.98	−8.5	−0.6	−28.2	LH	Limbic lobe, Uncus, BA 28
2.85	11.3	−9.4	−14	RH	Limbic lobe, Parahippocampal gyrus, BA34
2.79	21.2	9.1	−27.5	RH	Limbic lobe, uncus, BA 38
2.51	−48.5	−33.7	−23.6	LH	Temporal lobe, Fusiform gyrus, BA20
2.35	−58.5	−55	−17.6	LH	Temporal lobe, Fusiform gyrus, BA37
2.35	50.8	−0.6	−28.2	RH	Temporal lobe, Medial temporal gyrus, BA 21
2.34	−58.5	−8.7	−21.5	LH	Temporal lobe, Medial temporal gyrus, BA 20
1.90	1.5	−20.3	26.8	RH	Limbic lobe, Cingulate gyrus, BA23
1.53	1.5	23.4	22.2	RH	Limbic lobe, Anterior cingulate, BA24
1.19	40.9	2.4	29.4	RH	Frontal lobe, Inferior frontal gyrus, BA6
1.04	−38.5	2.4	29.4	LH	Frontal lobe, Inferior frontal gyrus, BA6
1.02	40.9	−30.4	34.9	RH	Parietal lobe, inferior parietal lobule, BA40

Tailarach coordinates (mm) corresponding to the intracranial generators explaining the difference voltages for related–unrelated proper names in the 300–380 ms time window, according to swLORETA (ASA) [23], grid spacing = 5 mm, estimated SNR = 3.

#### Fronto-central P/N400 (380–500 ms)

This ANOVA showed that the interaction lexical class x semantic associativeness was significant (F(1,13) = 5.55, p<0.05). Post-hoc comparisons indicated larger N400 amplitudes to common (−2.43 µV) than proper (0.2 µV) unrelated items (see values in the right part of Fig. 5), while no class differences whatsoever were found in response to related items (Tukey test, p = 0.72). The interaction associativeness x electrode was also significant (F(1,13) = 11.28, p<0.01), showing much larger N400 potentials to unrelated than related items, especially over the centroparietal region, which is the typical area for N400 scalp topography.

**Figure 7:**
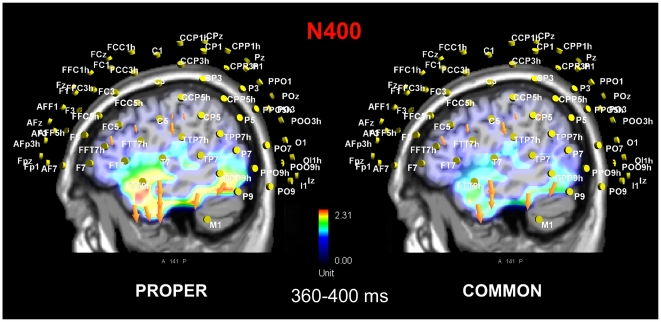
Sagittal view of N400 active sources for the contrast-unrelated minus related items for the two lexical class (Left: common, Right: proper), according to swLORETA, in the time window 360–400 ms.

In order to locate the possible neural source of the lexical effect for unrelated words, two separate swLORETA source reconstructions were performed on the difference-waves obtained by subtracting the ERPs to related common (or proper) nouns from those elicited by unrelated common (or proper) nouns in the time window 360–400 ms, representing the peak of N400 in the difference waves. The resulting neural activity, visible in Fig. 7, might reflect the activation of the neural circuits subserving semantic integration and retrieval processes for the two stimulus classes. The neural generators relative to this contrast, which included the left fusiform gyrus (BA20 and BA37), the right medial temporal gyrus (BA21) the right parahippocampal gyrus (BA34), the left and right inferior parietal lobule (BA40) and the left and right inferior frontal gyrus (BA6), were substantially identical, as shown in Fig. 7. This could be because, in the absence of specific episodic (personages) or semantic (things) information, and orthographical and phonological properties being equal, common and proper referents to nonexistent entities were very much alike in many respects.

## Discussion

### When memory retrieval is not required proper name semantic access (*conspecifcs knowledge*) is not more demanding

Behavioural data provide evidence that proper names have an advantage in the semantic relatedness decision task. Indeed, participants were asked to judge whether the second item of each pair was associated with the first in defining a well-recognized person or entity. Both accuracy and speed data showed that if semantic associativeness, written frequency and length were equal, the task was more effortful for common nouns. This hypothesis is supported by the N400 latency data, which show slower N400 potentials for unrelated common than proper nouns at all scalp sites. While previous studies on memory retrieval of proper vs. common nouns argued that common nouns are accessed faster than proper names [24] our data seem to suggest that, when memory retrieval is not required, semantic access is not necessarily more difficult for proper names. One other way to consider N400 latency data is observing that the unrelated proper noun response dropped off in amplitude earlier, resulting in an earlier peak than the common noun response. The interpretation of this pattern might reflect a longer continuation of the same process in the common noun case, or it might reflect an additional later process that is not invoked in the proper noun case.

The finding that RTs were faster to proper than common nouns was unexpected. Considering this along with previously published data [4], [13], [25] suggesting that the retrieval of purely referential information characterized by detailed spatio-temporal coordinates (proper names) is more demanding, we administered a further questionnaire *a posteriori* to determine the cloze probability of the second item of each pair of common and proper nouns. The cloze probability is defined as the percentage of individuals who continue a sentence fragment with that item in an offline sentence completion task [26]. For this purpose, the first item of each pair (in the form of a two-column list) was administered to a new group of 14 judges (5 men and 9 women) aged between 23 and 35 years, who had never seen the stimuli before. They were asked to write beside each noun and first name (randomly mixed with each other but presented in separate lists) a strongly related adjective (the first that came to mind) for common nouns, or a strongly related last name for proper names. The cloze probability of the second item in each pair was therefore computed by quantifying the percentage of judges who had actually selected the expected adjective or the last name of a famous person: a value of 1 meant that all the judges (100%) had chosen the experimental stimulus as the completing item of a pair, while 0 meant that none of the subjects had done so. We also performed an ANOVA across lexical classes (common and proper nouns) considering the cloze probability values as dependent variables. Statistical comparison showed that proper name pairs had a higher cloze probability than common nouns (F(1,188) = 7.11, p<0.01), with a value of 0.70 for proper names and 0.58 for common nouns. These results were interpreted in the light of the RT data as a possible causal explanation for the shorter RTs recorded in the semantic association task for proper than common nouns pairs. Indeed, it is possible that in a context of semantic priming, that is, by providing a semantic clue to activate a specific memory link, the uniqueness of proper name information promotes rather than retards the fast retrieval of the second item of a pair embodying a unique referent (e.g. ARNOLD SCHWARZENEGGER). In contrast, the conceptual unit represented by a common noun pair (e.g. “TRAFFIC OFFICER”) is more abstract and general. Looking it from another perspective, the unfamiliar proper noun and common noun pairs differed critically in semantic integration, in that adjective + noun pairs (e.g., *THORACIC SAW*) were at least sometimes compositionally interpreted (the meaning of each word must be combined) whereas in the unfamiliar proper noun case, there was no possible ‘interpretation’ (e.g., *GERRY PANTANI*). In this context the N400 amplitude data might reflect semantic integration difficulty (e.g. [16]).

A different pattern of results was obtained by Proverbio et al. [4], who reported slower RTs to proper than common nouns in a memory retrieval task. In that study, however, participants were asked to retrieve the phonological forms of proper and common nouns upon a written definition of their functional properties (e.g. Opera theatre in Milan = LA SCALA; Collects and sells old furniture = ANTIQUARIAN), while in the present study names were provided in full to the readers, thus allowing semantic and episodic information to be retrieved easily on the basis of orthographic inputs. The difference between lexical classes in the present experiment might also be related to the degree of semantic associativeness between the first and second items of a pair.

### Person names is more emotional and sensory vivid than common noun access

Analysis of the ERP data indicated strong lexical class and relatedness effects at both occipito/temporal and anterior sites as early as 300 ms post-stimulus. The analysis of posterior activity in the 300–380 ms time window showed a significant semantic relatedness effect (with larger P400 to related than unrelated items) and the presence of a much larger P400 to related proper than common nouns at occipito/temporal than temporo/parietal sites. The anatomical and functional dissociation observed for the amplitude of the posterior P4/N400 response, suggesting a role for the ventral visual pathway in the greater evoked response to name pairs denoting famous persons than recognized entities, led to the hypothesis that a further (unexplored) dimension might subtend different category-related functional properties. In particular, the hypothesis was advanced that the two lexical classes differed in terms of imagery value, also called imageability. In order to test this hypothesis, two different questionnaires (one for related proper names and the other for related common nouns) were administered, *a posteriori*, to a new group of 14 judges (4 men and 10 women) 19–30 years old, who were asked to establish the degree of imageability of each item on a 5-point scale (1 = not imaginable; 5 = highly imaginable). Imageability was defined as the rapidity (immediacy) and ease with which a given word evoked the corresponding mental image (of a person or a thing), either visual, auditory or supplied with any other sensory representation. Items were presented randomly mixed and the presentation order was different for each subject. Half the judges first filled in the questionnaire relative to proper names, and half relative to common nouns. The mean imagery value for each item across judges was then computed. The scores belonging to the two lexical classes underwent a one-way ANOVA, which showed that the lexical class factor was significant (F(1,188) = 30.08, p<0.01). Indeed, proper name pairs (4.47) proved on average to be more imaginable than common noun pairs (3.81). These results support the hypothesis that the greater activation of posterior brain regions (including the left fusiform gyrus and the right middle temporal gyrus) while recalling famous persons vs. real entities might reflect the larger amount of sensory association linked to proper names, and contribute to the greater imagery values ascertained for this category. In particular, it is likely that the images of famous faces might be automatically and vividly recalled to a greater extent than the visual images of more abstract entities such as “botanic garden”, “classical dance” or “cross-country race.” The fMRI observation that the left fusiform gyrus has a role in the processing of familiar faces (e.g. [27]) is consistent with this hypothesis. Also, the medial temporal cortex was found to be crucial for the processing of person names in a PET study by Gorno-Tempini and coworkers [5] and in an event-related fMRI study by Douville and coworkers [7], who also revealed the importance of the parahippocampal region and the hippocampal complex (including the medial temporal lobe) in recognizing recent and remote famous names. The involvement of the right parahippocampal region [28] and of the fusiform gyrus in semantic decision tasks involving famous faces was also demonstrated by Sergent and coworkers [29]. Overall, the role of the left temporal cortex in proper name retrieval (which, in our study, offered the strongest LORETA cortical generator in both the recognized persons minus things (BA21, 21) and the recognized minus unknown persons (BA20, 37) contrasts) seems to be a well established finding (e.g. [5], [30], [31], [32]).

As for the persons vs. things comparison, a LORETA was performed on the difference wave obtained by subtracting the ERPs to related common from proper nouns. This revealed a series of intracranial neural generators explaining the surface difference voltage, which included the limbic regions (right parahippocampal gyrus and right cingulate), possibly indicating a more emotional connotation of person than thing names; the right medial frontal regions, possibly involved in the retrieval of episodic information relevant to biographical aspects of the persons recalled; and temporal regions (such as the left fusiform gyrus and the right medial temporal gyrus), which might additionally depend on the greater imagery values of proper names. Alternately, a purely linguistic activation could be hypothesized, involving the Visual Word Form Area (left fusiform gyrus of the temporal cortex) being more responsive to (equally frequent) more salient or arousing words [33], [34]. Indeed, reading the names “Barack Obama” or “Julia Roberts” might be more emotional experiences than reading “Persian carpet” or “water vapour”, as also proved by the activation of limbic regions by the proper names. The combination of inferior parietal lobe (BA40) and inferior frontal lobe activation, also present in the contrast unrelated-related proper nouns (N400 to unknown vs. recognized persons), is strongly consistent with the finding of a PET study [6] investigating the retrieval of the visual representation of a face when presented with an associated name. The three main regions involved in this task proved to be located in the inferior frontal gyrus (BA 45), the medial frontal gyrus (BA 6) and the supramarginal gyrus of the inferior parietal lobe (BA 40), which were also found to be active in our study (see Table 2 and 3). These results indicate that the visual images of recognized proper names were actually retrieved during task performance, even if not required by the task.

### Proper vs. common nouns are not implemented differently in the brain as belonging to different grammatical classes

The other interesting finding is that at both anterior and posterior sites, in the 300–380 ms time window, and while P400 was markedly greater for processing of proper than common nouns, no class difference was manifest in the response to unrelated words. Only in the next latency range and only at anterior sites was a larger N400 observed to unrelated common than proper nouns, partly supporting the hypothesis that the semantic task for the latter category is easier. On the basis of the present data it can be proposed that is not the grammatical category (being a common or a proper noun) per se that determines a difference in neural processing of names, otherwise a proper/common noun difference should also be observable for unrelated pairs of first names/surnames vs. nouns/adjectives, which did not occur in the present study. Indeed, the linked non-verbal information is very likely to contribute to determining a different pattern of neural activity for representing famous actors or singers vs. semantic concepts defining things. It has been proposed that memory for proper names may share some of the properties that distinguish episodic from semantic memory at a peripheral, lexical level [4]). Indeed, episodic memory retrieval may be defined as the retrieval of unique information linked to precise spatio-temporal coordinates as opposed to concepts stored in semantic memory, which are independent of any spatial or temporal context. Therefore, retrieval of common and proper nouns might, in principle, differentially activate neurofunctional circuits of memory because of their intrinsic properties (i.e. proper names referring to unique individuals and common nouns being linked more abstract conceptual information). On the other hand, other models have hypothesized the existence of different domains for storing information concerning persons and things (e.g. the famous Bruce and Young model [35] as well as neurological [36], [37] and neuroimaging studies [8]). Our data provide evidence that unrelated common and proper nouns are treated quite similarly up to about 350–400 ms. And indeed word-pairs such as “culpable sign” and “Steven Carrisi” are similar from the orthographical and phonological points of view (since many perceptual factors were balanced for). If anything, it might easier to establish that a “Steven Carrisi” is unknown to us than that “culpable sign” is a nonexistent entity, as suggested by the larger N400 values for common unrelated nouns. But as for the neural generators of the N400 effect, no difference arose as a function of lexical class. Its also interesting to note that while the N400 effect (unrelated minus related) was larger over the left hemisphere, there was no hemispheric asymmetry for the P400 responses, probably indicating a more linguistic than representational neural activity. Further evidence of a substantial similarity in the neural processing of unrelated nouns comes from the LORETA source reconstruction performed for both proper and common nouns (unrelated minus related contrast) in the time window 360–400 ms, corresponding to the peak of the N400 response. The results indicate that the surface difference-voltage was explained by the same neural generators, namely the left fusiform gyrus (BA20 and BA37), the right medial temporal gyrus (BA21), the right parahippocampal gyrus (BA34), and the left and right inferior frontal (BA6). It is interesting to note that the left fusiform gyrus, the right medial temporal gyrus [34], [38] and the right parahippocampal gyrus [39] correspond to the structures that according to some electrophysiological and source localization studies are involved in semantic processing, and more generally in generating the N400 response to semantic incongruence. In particular, bilateral anterior medial temporal lobe structures are supposed to be strongly involved in semantic processing [40], [41], [42] and so are the inferior temporal lobe [43] and the anterior fusiform gyrus [41]. In the light of the notion that the N400 response would indicate semantic integration processes [16], the LORETA inverse solutions suggest a substantially similar process of integrating semantic information for words (orthographically and phonologically balanced) lacking semantic or episodic contents (i.e., unrelated proper and common nouns). Kutas and Federmeier [44] have also argued that one of the mental processes reflected by the N400 amplitude is search within the mental lexicon. In this context, the larger N400 amplitudes along with the later N400 latencies to common unrelated nouns, followed by slower RTs to the former class of items, might indicate a more difficult/lengthy search [45]. Overall, the difference in cloze probability could explain the pattern of reaction times and of N400 amplitude observed. Indeed, according to Kutas & Federmeier [44] cloze probability may drive differences in N400 amplitude, the smaller amplitude the more facilitated the access to semantic memory. One might object that many of the differences observed between common and proper noun stimuli in the study might be due to the differences between the stimuli in cloze probability, and could potentially mask differences or similarities in processing of these stimuli. However, the identification of an identical network of regions active during processing of unrelated pairs of the 2 categories supports our general claim that proper and common nouns are not differently implemented in the brain as belonging to different grammatical classes. Alternately, it cannot be completely excluded that the unrelated-related difference reflects a difference in post-access processing that is mediated by the same structures across common/proper nouns that are instead stored in different areas. However, we regard this interpretation as highly hypothetical.

### Conclusions

The aim of the present study was to investigate the existence of possible differences in the neurofunctional circuits subtending the processing of common vs. proper nouns, in a semantic decision rather than a memory retrieval task [4]. In order to test the ability to process information about person vs. thing names specifically, thus elucidating the storage of conceptual and episodic knowledge in the brain, nouns in the two categories were visually presented to the participants, so memory retrieval of the phonological forms of words (which is thought to be more effortful for purely referential expressions [11], [12], [13]) was not required.

Another important feature of this study was the effort to balance several aspects of the two lexical classes (proper, common) and stimulus types (related, unrelated): written frequency of use, length, semantic associativeness, familiarity. Furthermore, the cloze probability of common and proper noun pairs was measured a posteriori and compared statistically, showing that proper names actually had a higher cloze probability than common pairs (0.70 vs. 0.58). The imageability of each related name pair was also evaluated, and it turned out that proper names were more imageable (4.47) than common nouns (3.81). This result may probably explain a major difference from a previous ERP study ([4], reporting a larger occipito/temporal activation for common noun memory retrieval), where common nouns (not necessarily name pairs) represented more concrete items since they included many animals, natural events, plants, familiar objects and fruit names (e.g. banana, lemon, river, rose); nothing like “figure of speech” or “public opinion”.

The results of the present experiment showed that:

1. In a semantic decision task (associativeness), proper names are processed more quickly than common nouns. This pattern of results fits with the finding of slower anterior N400 latencies for unrelated common nouns and larger N400 amplitudes for the latter word pair types.
2. Apart from that, no difference whatsoever was found in the processing of unrelated common or proper nouns at any time range or scalp site. Furthermore, a LORETA performed on the neural generators of the N400 effect (unrelated minus related) revealed a striking similarity between the generators relevant to the two lexical classes. This lack of difference in the neural processing of unrelated common and proper noun pairs suggests that the two classes of words are not represented in grammatically-specific storages, but are treated similarly when there are no links to specific episodic or conceptual representations.
3. The finding of stronger brain activation over the occipito/temporal cortex for representing recognized persons (vs. unknown persons or recognized things) is consistent with the greater imagery values of the proper name pairs. The neural generators involved included the left fusiform gyrus, limbic and parahippocampal regions and the IP and IF areas, which are thought to be involved in the conjoined processing of a familiar face with the relevant episodic information. In addition, the left FG activation might indicate an effect of attentional modulation of VWFA activity for more salient/emotional stimuli (“Barack Obama” vs. “Joseph Bonamici”).

Overall, the present data do not support the hypothesis that category-specific effects are due to the existence of distinct semantic storages for proper and common nouns, but possibly to their affinity with episodic vs. semantic memory.

## Supporting Information

Appendix S1 (0.52 MB DOC)
