# Supplementary material for: How Are ‘Barack Obama’ and ‘President Elect’ Differentially Stored in the Brain? An ERP Investigation on the Processing of Proper and Common Noun Pairs
Source: PLoS One. 2009 Sep 23;4(9):e7126. doi: 10.1371/journal.pone.0007126 (PMC2741605; doi:10.1371/journal.pone.0007126)
Supplement: Appendix S1 — (0.52 MB DOC) [file pone.0007126.s001.doc]

**Appendix S1.**

**Pairs of semantically related proper names and last names**

| 1. WOODY | ALLEN |
| --- | --- |
| 1. CLAUDIO | AMENDOLA |
| 1. PAMELA | ANDERSON |
| 1. GIULIO | ANDREOTTI |
| 1. AMBRA | ANGIOLINI |
| 1. BIAGIO | ANTONACCI |
| 1. MANUELA | ARCURI |
| 1. DARIO | ARGENTO |
| 1. GIORGIO | ARMANI |
| 1. ROBERTO | BAGGIO |
| 1. PIPPO | BAUDO |
| 1. DAVID | BECKHAM |
| 1. MONICA | BELLUCCI |
| 1. OSAMA | BIN LADEN |
| 1. ROSY | BINDI |
| 1. MIKE | BONGIORNO |
| 1. PAOLO | BONOLIS |
| 1. PAOLO | BORSELLINO |
| 1. UMBERTO | BOSSI |
| 1. NANCY | BRILLI |
| 1. NAOMI | CAMPBELL |
| 1. FABIO | CANNAVARO |
| 1. MILLY | CARLUCCI |
| 1. PIERFERDINANDO | CASINI |
| 1. FIDEL | CASTRO |
| 1. ALESSANDRO | CECCHI PAONE |
| 1. ADRIANO | CELENTANO |
| 1. CARLO AZEGLIO | CIAMPI |
| 1. BILL | CLINTON |
| 1. GEORGE | CLOONEY |
| 1. MARTINA | COLOMBARI |
| 1. CRISTOFORO | COLOMBO |
| 1. DEBORA | COMPAGNONI |
| 1. TOM | CRUISE |
| 1. TOTO | CUTUGNO |
| 1. CHRISTIAN | DE SICA |
| 1. ALESSANDRO | DEL PIERO |
| 1. LEONARDO | DICAPRIO |
| 1. BARBARA | D'URSO |
| 1. THOMAS | EDISON |
| 1. NATALIA | ESTRADA |
| 1. ORIANA | FALLACI |
| 1. ROSARIO | FIORELLO |
| 1. GIUSEPPE | GARIBALDI |
| 1. RICHARD | GERE |
| 1. GENE | GNOCCHI |
| 1. IRENE | GRANDI |
| 1. EZIO | GREGGIO |
| 1. GIANLUCA | GRIGNANI |
| 1. ADOLF | HITLER |
| 1. DUSTIN | HOFFMAN |
| 1. SADDAM | HUSSEIN |
| 1. ENZO | IACHETTI |
| 1. KEVIN | COSTNER |
| 1. LUCA | LAURENTI |
| 1. LUCIANO | LIGABUE |
| 1. MARCELLO | LIPPI |
| 1. MASSIMO | LOPEZ |
| 1. SOFIA | LOREN |
| 1. NELSON | MANDELA |
| 1. ALESSIA | MARCUZZI |
| 1. VALERIA | MARINI |
| 1. FREDDIE | MERCURY |
| 1. DEMI | MOORE |
| 1. BENITO | MUSSOLINI |
| 1. GIANNA | NANNINI |
| 1. BARACK | OBAMA |
| 1. GIORGIO | PANARIELLO |
| 1. FEDERICA | PANICUCCI |
| 1. ALBA | PARIETTI |
| 1. PAOLA | PEREGO |
| 1. IRENE | PIVETTI |
| 1. RENATO | POZZETTO |
| 1. ELVIS | PRESLEY |
| 1. ROBERT | REDFORD |
| 1. JULIA | ROBERTS |
| 1. MASSIMILIANO | ROSOLINO |
| 1. STEFANIA | SANDRELLI |
| 1. MICHELE | SANTORO |
| 1. NICOLAS | SARKOZY |
| 1. MICHAEL | SCHUMACHER |
| 1. ARNOLD | SCHWARZENEGGER |
| 1. WILLIAM | SHAKESPEARE |
| 1. CLAUDIA | SCHIFFER |
| 1. BRITNEY | SPEARS |
| 1. SILVESTER | STALLONE |
| 1. UMA | THURMAN |
| 1. SIMONA | VENTURA |
| 1. CARLO | VERDONE |
| 1. BRUNO | VESPA |
| 1. DENZEL | WASHINGTON |
| 1. ROBBIE | WILLIAMS |
| 1. BRUCE | WILLIS |
| 1. KAROL | WOJTYLA |
| 1. IVA | ZANICCHI |

**Pairs of semantically unrelated proper names and last names**

| 1. ALBANO | AGNELLI |
| --- | --- |
| 1. ENZO | ALIGHIERI |
| 1. CLEMENTE | BAGLIONI |
| 1. WALTER | BANDERAS |
| 1. SILVIO | BARALE |
| 1. FLAVIO | BARDOT |
| 1. LEO | BATTISTI |
| 1. GIANNI | BENIGNI |
| 1. EROS | BERLUSCONI |
| 1. ANNA | BERTINOTTI |
| 1. DIEGO ARMANDO | BIAGI |
| 1. OSCAR LUIGI | BIGNARDI |
| 1. JOSEPH | BONAMICI |
| 1. AL | BRIATORE |
| 1. GIOVANNI | CANALIS |
| 1. CLAUDIO | CAPONE |
| 1. RAIMONDO | CARREY |
| 1. STEVEN | CARRISI |
| 1. ROMANO | CAVALLI |
| 1. FABRIZIO | CHECHI |
| 1. DANTE | CIPOLLINI |
| 1. MICHELLE | CLERICI |
| 1. MARIO | CONNERY |
| 1. LUCIO | COSTANZO |
| 1. FILIPPO | CRAXI |
| 1. PAOLO | CUCCARINI |
| 1. YURI | D'ALEMA |
| 1. ALBERT | DE FILIPPI |
| 1. UGO | EINSTEIN |
| 1. GIACOMO | FALCHI |
| 1. LAURA | FALCONE |
| 1. ANTONELLA | FALETTI |
| 1. PAOLA | FERILLI |
| 1. MARILIN | FORMIGONI |
| 1. JOHN FITZGERALD | FOSCOLO |
| 1. GIANNI | FRACCI |
| 1. GIORGIO | FREEMAN |
| 1. ALBERTO | FREUD |
| 1. MORGAN | FRIZZI |
| 1. AIDA | GALEAZZI |
| 1. ALBERTO | GIBSON |
| 1. JOHN | GRILLO |
| 1. MAURIZIO | GULLOTTA |
| 1. ROBERTO | HUNZIKER |
| 1. UMBERTO | INCONTRADA |
| 1. MEL | INZAGHI |
| 1. RITA | JOLIE |
| 1. PAOLO | KENNEDY |
| 1. MASSIMO | LENNON |
| 1. VITTORIO | LEOPARDI |
| 1. MARCO | LEVI MONTALCINI |
| 1. LEONARDO | LOLLOBRIGIDA |
| 1. NANNI | MALDINI |
| 1. MARIA | MAMMUCCARI |
| 1. GIAMPIERO | MANZONI |
| 1. VALENTINO | MARADONA |
| 1. VANESSA | MASTELLA |
| 1. ELISABETTA | MENTANA |
| 1. TEO | MONROE |
| 1. CRISTINA | MORANDI |
| 1. BRIGITTE | MORATTI |
| 1. CHUCK | MORETTI |
| 1. CARLA | MORRICONE |
| 1. SEAN | NAPOLITANO |
| 1. GIORGIO | NORRIS |
| 1. GERRY | PANTANI |
| 1. JIM | PAOLI |
| 1. SIGMUND | PARODI |
| 1. GIUSEPPE | PAUSINI |
| 1. ROBERTO | PAVAROTTI |
| 1. GINA | PEZZALI |
| 1. FAUSTO | PICASSO |
| 1. PABLO | PIERACCIONI |
| 1. ENNIO | PRODI |
| 1. TEO | RAMAZZOTTI |
| 1. ALENA | RATZINGER |
| 1. CESARA | ROSSI |
| 1. GINO | SCALFARO |
| 1. MAX | SCOTTI |
| 1. ROBERTO | SEREDOVA |
| 1. LETIZIA | SGARBI |
| 1. LORELLA | SINATRA |
| 1. SABRINA | SORDI |
| 1. ENRICO | SPIELBERG |
| 1. FRANK | SPOSINI |
| 1. BEPPE | STONE |
| 1. ALESSANDRO | TEOCOLI |
| 1. ANTONIO | TOMBA |
| 1. LUCIANO | TRAVOLTA |
| 1. DARIA | VELTRONI |
| 1. SHARON | VERDI |
| 1. ANGELINA | VERONESI |
| 1. BETTINO | VIANELLO |
| 1. LAMBERTO | VILLAGGIO |
| 1. JOHN | YESPICA |

**Pairs of semantically related common nouns and adjectives**

| 1. UOVO | SODO |
| --- | --- |
| 1. PESO | NETTO |
| 1. OPERA | LIRICA |
| 1. RISTORANTE | CINESE |
| 1. RACCORDO | ANULARE |
| 1. PSICOLOGIA | CLINICA |
| 1. TRIANGOLO | ISOSCELE |
| 1. GIARDINO | BOTANICO |
| 1. DANZA | CLASSICA |
| 1. CORSA | CAMPESTRE |
| 1. ELEZIONI | POLITICHE |
| 1. GIUDIZIO | UNIVERSALE |
| 1. GAZZA | LADRA |
| 1. INSALATA | RUSSA |
| 1. PIANTA | GRASSA |
| 1. IMPERO | ROMANO |
| 1. IGIENE | DENTALE |
| 1. CORTE | SUPREMA |
| 1. TUBO | CATODICO |
| 1. ERA | GLACIALE |
| 1. INSURREZIONE | POPOLARE |
| 1. ERUZIONE | VULCANICA |
| 1. URANIO | IMPOVERITO |
| 1. TRASMISSIONE | TELEVISIVA |
| 1. MIDOLLO | OSSEO |
| 1. SEMAFORO | ROSSO |
| 1. PASSATO | REMOTO |
| 1. SETTIMANA | BIANCA |
| 1. NASTRO | ADESIVO |
| 1. PREDICATO | VERBALE |
| 1. MIMICA | FACCIALE |
| 1. FIGURA | RETORICA |
| 1. OPINIONE | PUBBLICA |
| 1. COMPUTER | PORTATILE |
| 1. AGGETTIVO | POSSESSIVO |
| 1. CELLA | FRIGORIFERA |
| 1. IMPRESA | EDILE |
| 1. VAPORE | ACQUEO |
| 1. BIANCHERIA | INTIMA |
| 1. GALLO | CEDRONE |
| 1. TUMORE | MALIGNO |
| 1. PECCATO | MORTALE |
| 1. FORESTA | PLUVIALE |
| 1. REALTA' | VIRTUALE |
| 1. PAPILLA | GUSTATIVA |
| *HARD-BOILED* | *EGG* |
| *NET* | *WEIGHT* |
| *LYRICAL* | *OPERA* |
| *CHINESE* | *RESTAURANT* |
| *RING* | *ROAD* |
| *CLINICAL* | *PSYCHOLOGY* |
| *ISOSCELES* | *TRIANGLE* |
| *BOTANIC* | *GARDEN* |
| *CLASSICAL* | *DANCE* |
| *CROSS-COUNTRY* | *RACE* |
| *GENERAL* | *ELECTIONS* |
| *LAST* | *JUDGEMENT* |
| *(THIEF)* | *MAGPIE* |
| *RUSSIAN* | *SALAD* |
| *FAT* | *PLANT* |
| *ROMAN* | *EMPIRE* |
| *DENTAL* | *HYGIENE* |
| *SUPREME* | *COURT* |
| *CATHODIC* | *TUBE* |
| *GLACIAL* | *AGE* |
| *PEOPLE’S* | *INSURRECTION* |
| *VOLCANIC* | *ERUPTION* |
| *DEPLETED* | *URANIUM* |
| *TELEVISION* | *TRANSMISSION* |
| *BONE* | *MARROW* |
| *RED* | *LIGHT* |
| *REMOTE* | *PAST* |
| *SKI* | *HOLIDAY* |
| *SCOTCH* | *TAPE* |
| *VERB* | *PHRASE* |
| *FACIAL* | *MIMICRY* |
| *FIGURE* | *OF SPEECH* |
| *PUBLIC* | *OPINION* |
| *PORTABLE* | *COMPUTER* |
| *POSSESSIVE* | *ADJECTIVE* |
| *REFRIGERETING* | *ROOM* |
| *PROPERTY* | *DEVELOPER* |
| *WATER* | *VAPOUR* |
| */* | *UNDERWEAR* |
| *GROUSE* | *(COCK)* |
| *MALIGNANT* | *TUMOR* |
| *DEADLY* | *SIN* |
| *RAIN* | *FOREST* |
| *VIRTUAL* | *REALITY* |
| *TASTE* | *BUD* |

| 1. OLIO | VEGETALE |
| --- | --- |
| 1. INVASIONE | BARBARICA |
| 1. PARADISO | TERRESTRE |
| 1. MARTELLO | PNEUMATICO |
| 1. MACCHINA | FOTOGRAFICA |
| 1. BAGNO | TURCO |
| 1. CARTA | VELINA |
| 1. MATERIA | GRIGIA |
| 1. ALCOOL | ETILICO |
| 1. CARRO | FUNEBRE |
| 1. INVESTIGATORE | PRIVATO |
| 1. TAPPETO | PERSIANO |
| 1. CHIRURGIA | ESTETICA |
| 1. SALICE | PIANGENTE |
| 1. GUIDA | TURISTICA |
| 1. FILO | CONDUTTORE |
| 1. SHOCK | ANAFILATTICO |
| 1. ESAME | ORALE |
| 1. ENCICLICA | PAPALE |
| 1. VIGILE | URBANO |
| 1. AURORA | BOREALE |
| 1. GUARDIA | GIURATA |
| 1. PREVIDENZA | SOCIALE |
| 1. SEDIA | GIREVOLE |
| 1. NAVICELLA | SPAZIALE |
| 1. EMBOLIA | POLMONARE |
| 1. GINNASTICA | ARTISTICA |
| 1. PORTA | SCORREVOLE |
| 1. POLIZZA | ASSICURATIVA |
| 1. SCACCO | MATTO |
| 1. FREDDO | POLARE |
| 1. SCALA | MOBILE |
| 1. FORZA | MOTRICE |
| 1. BOMBA | ATOMICA |
| 1. GHIANDOLA | SALIVARE |
| 1. PARETE | ROCCIOSA |
| 1. CAFFE' | CORRETTO |
| 1. ACETO | BALSAMICO |
| 1. RELIGIONE | CATTOLICA |
| 1. SEGRETERIA | TELEFONICA |

| *VEGETABLE* | *OIL* |
| --- | --- |
| *BARBARIC* | *INVASION* |
| *EARTHLY* | *PARADISE* |
| *HAMMER* | *DRILL* |
| *PHOTOGRAPHIC* | *CAMERA* |
| *TURKISH* | *BATH* |
| *TISSUE* | *PAPER* |
| *GRAY* | *MATTER* |
| *ETHYLIC* | *ALCOHOL* |
| */* | *HEARSE* |
| *PRIVATE* | *INVESTIGATOR* |
| *PERSIAN* | *CARPET* |
| *COSMETIC* | *SURGERY* |
| *WEEPING* | *WILLOW* |
| *TOURIST* | *GUIDE* |
| */* | *LEITMOTIV* |
| *ANAPHYLACTIC* | *SHOCK* |
| *ORAL* | *EXAMINATION* |
| *PAPAL* | *ENCYCLICAL* |
| *TRAFFIC* | *OFFICER* |
| *POLAR* | *LIGHTS* |
| *SECURITY* | *GUARD* |
| *SOCIAL* | *SECURITY* |
| *SWIVEL* | *CHAIR* |
| *SPACE* | *SHUTTLE* |
| *PUMONARY* | *EMBOLISM* |
| *ARTISTIC* | *GYMNASTICS* |
| *SLIDING* | *DOOR* |
| *INSURANCE* | *POLICY* |
| *CHECK* | *MATE* |
| *COLD* | *WEATHER* |
| *SLIDING* | *SCALE (ESCALATOR)* |
| *MOTIVE* | *POWER* |
| *ATOMIC* | *BOMB* |
| *SALIVARY* | *GLAND* |
| *ROCK* | *FACE* |
| *LACED* | *COFFEE* |
| *AROMATIC* | *VINEGAR* |
| *CATHOLIC* | *RELIGION* |
| *ANSWERING* | *MACHINE* |

| 1. ANIMALE | DOMESTICO |
| --- | --- |
| 1. OPERAZIONE | CHIRURGICA |
| 1. ITER | LEGISLATIVO |
| 1. PARENTESI | TONDA |
| 1. PATATINA | FRITTA |
| 1. GRAZIA | DIVINA |
| 1. PANNA | MONTATA |
| 1. SCI | NAUTICO |
| 1. DIARIO | SCOLASTICO |
| 1. SCLEROSI | MULTIPLA |
| *DOMESTIC* | *ANIMAL* |
| *SURGICAL* | *OPERATION* |
| *LEGISLATIVE* | *PROCEDURE* |
| *ROUND* | *BRACKET* |
| *FRENCH* | *FRY* |
| *SAVING* | *GRACE* |
| *WHIPPED* | *CREAM* |
| *WATER* | *SKI* |
| *SCHOOL* | *DIARY* |
| *MULTIPLE* | *SCLEROSIS* |

**Pairs of semantically unrelated common nouns and adjectives.**

| 1. ARTI | BLU |
| --- | --- |
| 1. CEMENTO | SANTO |
| 1. INIEZIONE | NAVALE |
| 1. SALE | PENALE |
| 1. CAMPO | CESAREO |
| 1. ANGELO | CHIMICO |
| 1. CARTONE | COMPRESSORE |
| 1. FRUTTA | VIVENTE |
| 1. ORSO | STRADALE |
| 1. COLONNA | SATANICA |
| 1. CORPO | ALFABETICO |
| 1. ATTORE | GENEALOGICO |
| 1. BOOM | VIVO |
| 1. FEDE | SECCA |
| 1. ACIDO | EROICO |
| 1. SEGA | TORACICA |
| 1. CORRENTE | PASTICCERA |
| 1. FEDINA | ACUSTICA |
| 1. PRINCIPE | TRAVERSO |
| 1. STRISCE | MARZIALI |
| 1. CAPRO | MUSICALE |
| 1. DISTROFIA | METALLICA |
| 1. ALBERO | DENTISTICO |
| 1. SISTEMA | SCIATICO |
| 1. QUOZIENTE | RETTO |
| 1. FALDA | MEDIA |
| 1. ACQUA | ETNICA |
| 1. ARGENTO | GROSSO |
| 1. PERITO | PORTANTE |
| 1. SPORT | POSTALE |
| 1. CANTIERE | ZODIACALE |
| 1. ANNO | BATTUTO |
| 1. AFASIA | MONDIALE |
| 1. CATENA | TRIENNALE |
| 1. MALATTIA | CADENTE |
| 1. RICETTA | FERROVIARIA |
| 1. BARRIERA | COTTA |
| 1. PROSCIUTTO | COMICO |
| 1. CIOCCOLATO | UMANO |
| 1. TELEFONO | NERVINO |
| 1. CARTELLO | COLPOSO |
| 1. ANGOLO | NERVOSO |
| 1. SEGNO | IDROFILO |
| 1. CHITARRA | CAPITALE |
| 1. LEGA | INFETTIVA |
| *BLUE* | *ARTS* |
| *SAINT* | *CEMENT* |
| *NAVAL* | *INJECTION* |
| *PENAL* | *SALT* |
| *CAESAREAN* | *FIELD* |
| *CHEMICAL* | *ANGEL* |
| *STEAM* | *CARDBOARD* |
| *LIVING* | *FRUIT* |
| *ROAD* | *BEAR* |
| *SATANIC* | *COLUMN* |
| *ALPHABETIC* | *BODY* |
| *GENEALOGIC* | *ACTOR* |
| *ALIVE* | *FLUSH* |
| *DRIED* | *FAITH* |
| *HEROIC* | *ACID* |
| *THORACIC* | *SAW* |
| *CUSTARD* | *CURRENT* |
| *ACOUSTIC* | *CRIMINAL RECORD* |
| *CROSSWISE* | *PRINCE* |
| *MARTIAL* | *CROSSING* |
| *MUSICAL* | *GOAT* |
| *METALLIC* | *DYSTROPHY* |
| *DENTAL* | *TREE* |
| *SCIATIC* | *SYSTEM* |
| *RIGHT* | *QUOTIENT* |
| *AVERAGE* | *STRATUM* |
| *ETHNIC* | *WATER* |
| *BIG* | *SILVER* |
| *ALL-BEARING* | *SURVEYOR* |
| *POSTAL* | *SPORT* |
| *ZODIACAL* | *CONSTRUCTION SITE* |
| *WROUGHT* | *YEAR* |
| *WORLDWIDE* | *APHASIA* |
| *THREE-YEAR* | *CHAIN* |
| *FALLING* | *DISEASE* |
| *RAILWAY* | *RECIPE* |
| *BAKED* | *BARRIER* |
| *COMIC* | *HAM* |
| *HUMAN* | *CHOCOLATE* |
| *NERVE* | *TELEPHONE* |
| *CULPABLE* | *SIGN* |
| *NERVOUS* | *ANGLE* |
| *ABSORBENT* | *MARK* |
| *CAPITAL* | *GUITAR* |
| *INFECTIVE* | *LEAGUE* |

| 1. MEDICO | MINATO |
| --- | --- |
| 1. LAUREA | ACQUIFERA |
| 1. TRAUMA | CUSTODE |
| 1. SALMONE | ANIMATO |
| 1. ORDINE | AZZURRO |
| 1. CASCHI | PEDONALI |
| 1. RIMESSA | MAGICA |
| 1. GRUPPO | BISESTILE |
| 1. OMICIDIO | CELLULARE |
| 1. FERRO | ESPIATORIO |
| 1. CREMA | GIUDIZIARIA |
| 1. STAZIONE | CIECA |
| 1. RULLO | SENILE |
| 1. POEMA | AFFUMICATO |
| 1. SLALOM | INTELLETTIVO |
| 1. CRIMINALITA' | NUZIALE |
| 1. PRESEPE | PESANTE |
| 1. RADICE | LEGALE |
| 1. INCHIESTA | MINERALE |
| 1. STUDIO | MURIATICO |
| 1. POSTA | MUSCOLARE |
| 1. PERA | UMANITARIA |
| 1. DISCO | BANCARIO |
| 1. SPIRITO | CRUDO |
| 1. BONIFICO | LETALE |
| 1. GAS | ARMATO |
| 1. BOLLETTINO | OCULARE |
| 1. SCUOLA | VOLANTE |
| 1. VIZIO | FONDENTE |
| 1. GUERRA | QUADRATA |
| 1. STELLA | LIMITATA |
| 1. ONDA | ORGANIZZATA |
| 1. SETTA | ELETTRICA |
| 1. FOSSA | OPERATORIA |
| 1. DROGA | LATERALE |
| 1. SCALA | CORALLINA |
| 1. TESTIMONE | CIRCOLARE |
| 1. MOSCA | MEDICA |
| 1. GABBIA | FLUENTE |
| 1. GESTO | BRUNO |
| 1. ILLUSIONE | BIOLOGICA |
| 1. NERVO | ECONOMICO |
| 1. MISSIONE | ELETTRONICA |
| 1. COTONE | EPICO |
| 1. MINORANZA | OTTICA |
| 1. DEMENZA | SONORA |
| 1. PARTO | CRANICO |
| *MINED* | *DOCTOR* |
| *WATERED* | *DEGREE* |
| *GUARDIAN* | *TRAUMA* |
| *ANIMATED* | *SALMON* |
| *AZURE* | *ORDER* |
| *PEDESTRIAN* | *HELMETS* |
| *MAGICAL* | *LINE OUT* |
| *LEAP* | *GROUP* |
| *CELLULAR* | *HOMICIDE* |
| *WHIPPING* | *IRON* |
| *JUDICIAL* | *CREAM* |
| *BLIND* | *STATION* |
| *SENILE* | *ROLLER* |
| *SMOKED* | *POEM* |
| *INTELLECTUAL* | *SLALOM* |
| *NUPTIAL* | *RAQUET* |
| *HEAVY* | *CRECHE* |
| *LEGAL* | *ROOT* |
| *MINERAL* | *INQUIRY* |
| *MURIATIC* | *STUDIO* |
| *MUSCULAR* | *MAIL* |
| *HUMANITARIAN* | *PEAR* |
| *BANKING* | *DISK* |
| *RAW* | *SPIRIT* |
| *LETHAL* | *TRANSFER* |
| *ARMED* | *GAS* |
| *OCULAR* | *BULLETTIN* |
| *FLYING* | *SCHOOL* |
| *MELT* | *VICE* |
| *SQUARED* | *WAS* |
| *LIMITED* | *STAR* |
| *ORGANIZED* | *WAVE* |
| *ELECTRIC* | *SECT* |
| *OPERATING* | *PIT* |
| *LATERAL* | *DRUG* |
| *CORALLINE* | *STAIR* |
| *CIRCULAR* | *WITNESS* |
| *MEDICAL* | *FLY* |
| *FLUENT* | *GAGE* |
| *BROWN* | *GESTURE* |
| *BIOLOGICAL* | *ILLUSION* |
| *ECONOMIC* | *NERVE* |
| *ELECTRONIC* | *MISSION* |
| *EPIC* | *COTTON* |
| *OPTICAL* | *MINORITY* |
| *RESONANT* | *DEMENTIA* |
| *CRANIAL* | *CHILDBIRTH* |

| 1. FORMULA | GIGANTE |
| --- | --- |
| 1. FLAUTO | ESTREMO |
| 1. EDIZIONE | MONTUOSA |

| *GIANT* | *FORMULA* |
| --- | --- |
| *EXTREME* | *FLUTE* |
| *MOUNTAINOUS* | *EDITION* |
